# The relation between perceived non-native features in the L1 speech of English migrants to Austria and their phonetic manifestation in L1 productions

**DOI:** 10.1177/13670069231217595

**Published:** 2024-01-13

**Authors:** Sanne Ditewig, Ulrich Reubold, Robert Mayr, Ineke Mennen

**Affiliations:** University of Graz, Austria; Cardiff Metropolitan University, UK; University of Graz, Austria

**Keywords:** L1 phonetic attrition, L1 attrition of speech, cross-linguistic influences, perceived non-nativeness, foreign accent, first language attrition of speech, non-native features, segmental features, prosodic features

## Abstract

**Aims:**

The purpose of this research was to investigate to what extent the most commonly identified non-native features in the L1 speech of late consecutive bilinguals are reflected in differences in the bilinguals’ productions of these features compared with those of monolingual speakers of the L1.

**Design:**

We investigated the L1 accent of English migrants to Austria and monolingual English speakers in the United Kingdom in two inter-related studies.

**Data:**

In Study 1, an accent-perception experiment, native English listeners rated the nativeness of the monolinguals’ and bilinguals’ L1 English productions of read sentences, using a 6-point scale, and subsequently commented on the accentual features associated with perceived non-nativeness. In Study 2, the monolinguals’ and bilinguals’ productions of the most commonly identified non-native features from Study 1 were compared acoustically and auditorily.

**Findings:**

The accent-perception experiment revealed significantly higher non-nativeness ratings for the bilinguals than the monolinguals. These were associated with a wide range of segmental and prosodic features (*n* = 836 feature tokens), with /iː/ and /ɪ/ the most commonly identified for segments, and articulation rate and intonation for prosody. The phonetic analysis of these features in Study 2, in turn, revealed that the bilinguals produced /ɪ/ closer to /iː/ than the monolinguals and had more final rises in questions, with the articulation rate differences between them just failing to reach significance.

**Originality:**

This is the first study to document a direct link between the features perceived as non-native in bilinguals’ L1 speech and their measurable manifestation in non-native L1 speech productions.

**Significance:**

This research contributes significantly to our understanding of the relationship between the perception and production of attrition in L1 speech, and shows that where listeners are able to provide consistent, detailed descriptions of the features associated with non-nativeness, they truthfully reflect measurable patterns in the speech signal.

## Introduction

Individuals have great difficulties learning the pronunciation of a second language (L2), especially when learning starts in adulthood. It is now well established that the L2 accent of adult sequential bilinguals is likely to be influenced by the speaker’s first language (L1) pronunciation, and learners typically end up with a noticeable foreign accent (see Wayland, 2021 for an overview). More recently, research has started to acknowledge that pronunciation influences are in fact bidirectional in nature, such that not only L1 influences affect L2 pronunciation, but L1 pronunciation can also change under the influence of the L2 (e.g., de Leeuw et al., 2012, 2013; Mayr et al., 2012; Stoehr et al., 2017). The process of changes to the L1 observed in the pronunciation of adult sequential bilinguals who are long-term immersed in an L2 environment is often referred to as ‘phonetic attrition’, and the resulting speech as ‘attrited’ (e.g., de Leeuw et al., 2013; Major, 2010; Mennen et al., 2022).

There is a large body of evidence supporting bidirectional influences in pronunciation, with reports of both L1 effects on L2 speech and L2-induced effects on L1 speech (see Wayland, 2021 and below for overviews). One of the most influential models on L2 acquisition of speech, the speech learning model (SLM) (Flege, 1995) and its recent revision, the SLM-*r* (Flege & Bohn, 2021) – a model that is frequently adopted to explain phonetic L1 attrition (e.g., Bergmann et al., 2016; de Leeuw et al., 2018; Mayr et al., 2012, 2020a) – also embraces the notion of bidirectionality. The SLM posits that the sound systems of bilinguals are not isolated from each other, but co-exist in a shared phonetic space. This co-existence leads to mutual interactions between the speaker’s language systems that can be assimilatory (where the L1 and L2 sounds shift towards each other, resulting in a merging of L1 and L2 sounds) or dissimilatory (where L1 and L2 sounds shift away from one another) in nature, with both types of interaction resulting in differences from the patterns found in monolinguals.

Changes to pronunciation in the L1 of late sequential bilinguals are well documented for prosodic and segmental features. For segments, L2-induced changes have been reported for plosives (e.g., Alharbi et al., 2022; Flege & Hillenbrand, 1984; Kornder & Mennen, 2021; Mayr et al., 2012; Stoehr et al., 2017), vowels (e.g., Bergmann et al., 2016; Kornder & Mennen, 2021; Mayr et al., 2012), fricatives (Dmitrieva et al., 2010; Reubold et al., 2021), rhotics (Ulbrich & Ordin, 2014), and laterals (Bergmann et al., 2016; de Leeuw et al., 2013, 2018; Reubold et al., 2021). Less is known about prosodic features, but changes have so far been found in intonation patterns (Mennen et al., 2022) and how they are phonetically realised in terms of their timing or pitch range (e.g., de Leeuw, 2019; de Leeuw et al., 2012; Mennen, 2004; Mennen et al., 2022). These studies show that immersion in an L2-speaking environment may often result in changes to L1 pronunciation and that such changes can occur in a wide range of segmental and prosodic features. Listeners show remarkable sensitivity to speech patterns that differ from those used in their speech community (Flege, 1984) and may perceive long-term L2-immersed bilinguals as sounding non-native in their L1 (e.g., Bergmann et al., 2016; de Leeuw et al., 2010; Hopp & Schmid, 2013), presumably based on features of their speech production that differed from the ones they themselves might use.

The latter claim, however, remains to be established directly. To date, only two studies have attempted to identify what specific features listeners associate with non-nativeness in L1 speech (Bergmann et al., 2016; Mayr et al., 2020a), and a further study has aimed to identify differences in accentual features between bilingual and monolingual speakers in a language contact situation (Mayr et al., 2020b). Mayr et al. (2020) obtained global accentedness ratings of L1 Spanish speakers of L2 English living in the United Kingdom by presenting listeners with 15-second stretches of semi-spontaneous speech (extracted from a picture-based narrative) and asking them to rate the speech samples for their perceived nativeness. Most importantly for the present paper, for samples rated ‘non-native’, listeners were asked to comment on what made the sample sound non-native to them. This resulted in 100 useable comments (some comments were too general to be used for further analysis), 65% of which were on segments and 35% on prosody. The authors conclude that listeners use both segments and prosody when judging a speaker’s native-speaker status, although they appear to base their judgements more on segments than on prosody. While this gives a good indication as to the features listeners associate with non-native speech, it remains unclear to what extent the impression of non-nativeness can be ascribed to differences from monolingual L1 productions, given that the speech samples were not analysed acoustically. The second relevant study is by Bergmann et al. (2016), which not only obtained global foreign-accent ratings (FARs) of the L1 speech of German speakers of English as an L2 living in North America but also conducted acoustic analyses of selected L1 speech samples. In particular, three vowels (/aː/, /ɛ/, /ɔ/) and the lateral /l/ from the speech samples used in their FAR study were analysed by means of formant analyses. For each speaker, it was determined how much their formants differed from those of monolingual L1 speakers. These acoustic distances to monolingual L1 productions were then compared to the speakers’ FARs. The results showed that those speakers whose speech differed most from monolingual L1 speakers were not the ones who received the highest FARs. This suggests that listeners might not base their nativeness judgements on the features that were analysed acoustically. Since the listeners in their study based their accent ratings on speech samples with a 20-second duration, of which only four sounds were acoustically analysed, it would have been surprising if a relation between these particular sounds and listener judgements had been found. Listeners’ judgements could equally well have been influenced by other segmental or prosodic differences from monolingual L1 speech than the ones that were analysed, or may have resulted from an accumulation of accentual patterns (e.g., Jilka, 2000; Ulbrich & Mennen, 2016). Finally, Mayr et al. (2020b) investigated accent perception in a language contact situation in South-West Wales. In this study, listeners from Wales rated short English speech samples from monolingual speakers from Wales and Welsh-English bilinguals on whether they thought the speakers in the sample were able to speak Welsh. Listeners were then asked what accentual features had led them to this conclusion. These features were phonetically analysed. Results showed that the listeners were able to distinguish bilinguals from monolinguals on the basis of their accents in English. Some of the accent features that the listeners considered characteristic of the monolingual and bilingual speakers’ English accents were found to correspond to differences between the actual samples that were rated by the listeners. This suggests that listeners are able to use perceptible differences in the speech patterns of monolingual and bilingual speakers to evaluate an individual’s accent.

Overall, the literature shows that L1 attrition of speech is widespread and that such changes can lead to the perception of a non-native accent in the L1. However, it remains largely unknown what underlies the perception of accentedness in the L1 and which features listeners perceive as non-native in the L1 of adult sequential bilinguals. Similarly, we know virtually nothing about the link between listeners’ impression of non-nativeness and changes occurring in a speaker’s L1 pronunciation. For instance, we do not know whether those features that are perceived as non-native by listeners correspond to actual differences in production from monolingual speakers of the L1. While changes in L1 pronunciation are not necessarily negative and to be avoided, it is important to address the above issues, as perceptible changes to an individual’s L1 accent may have important social consequences, because people tend to disfavour those who are perceived as different (e.g., Lippi-Green, 1997; Munro, 2003). Therefore, the aim of the current paper is to investigate which features listeners perceive as non-native in the L1 speech of adult sequential bilinguals (Study 1) and analyse whether and how the most frequently mentioned features differ from those produced by monolingual speakers of the L1 (Study 2). This way, a more direct link can be made between accent ratings, the features that listeners associate with non-native speech, and how these features are realised in the speech samples that listeners are basing their judgements on. This is investigated in a group of native speakers of Standard Southern British English (SSBE) who moved to Austria after puberty and have Austrian German as their L2.

## Study 1: accent rating and perceived non-native features

The purpose of Study 1 was to determine which features are perceived by monolingual listeners as non-native in the L1 speech of adult sequential bilinguals. To answer this question, and following Mayr et al. (2020a, 2020b), we conducted an accent-rating experiment with monolingual listeners from the United Kingdom, in which we asked them to categorise a speaker as native or non-native, indicate their level of confidence in their judgement and comment on the features that made samples sound non-native to them.

### Speakers

Two groups of speakers were included in this study: (1) late-sequential English–Austrian German bilinguals (*N* = 8, 4 females, 4 males), who were raised as monolingual speakers of SSBE and emigrated to Austria after puberty where they acquired Austrian German as their L2; and (2) SSBE-speaking controls in England (*N* = 3, 1 female, 2 males), who had never lived outside of England and reported foreign-language knowledge no higher than high-school level. Both groups were educated to tertiary level and there were no significant differences in average age between the groups (mean 45.6 years; Wilcoxon rank sum exact test *W* = 16, *p* = .497). The bilinguals varied in their length of residence (LoR) (average LoR: 18 years, range 3–37 years), age of arrival in the host country (AoA) (average 31 years, range 21–58 years), and use of L1 (average: 40.8%, range 23.3%–66.6%) and L2 (average: 59.2%, range 33.3%–77.5%).^1^ They were recruited through existing contacts, and by advertising in expat groups, the British Embassy, and language schools. The monolingual group of SSBE speakers was recruited through colleagues at universities in the United Kingdom.

### Materials and listeners

The to-be-rated speech samples were taken from an existing corpus of read speech, recorded online through WikiSpeech (Draxler & Jänsch, 2008), consisting of 313 carefully controlled English materials designed to contain potential sources of attrition in a range of segmental and prosodic features (cf. Mennen et al., 2022; Reubold et al., 2021 for details). From this corpus, we selected a set of 12 sentences per speaker to be used as speech samples in the accent-rating experiment, yielding a total of 12 (sentences) × 11 (speakers) = 132 speech samples. These samples were chosen as they incorporated a variety of sentence types, including statements and questions, and were thus expected to elicit a variety of intonation patterns along with a variety of segments (for instance, rhotics, laterals, and vowels). The number of speech samples used in the accent-rating experiment was determined by the need to get as many useable samples while avoiding fatigue effects, which may result in a decrease in accuracy of listener ratings (Schmid & Hopp, 2014; Schwarz et al., 2016). The number of monolingual speakers presented in the accent-rating experiment was kept deliberately low (i.e., 3 monolingual as opposed to 8 bilingual speakers) to avoid so-called ‘range effects’, where including many samples from native speakers may lead to an overestimation of even small differences from native patterns and as a result individuals with a slight accent may be perceived as heavily accented (Schmid & Hopp, 2014). The selected stimuli were then prepared for the accent-rating experiment by normalising their amplitude to −3 dB and sampling size to 16 kHz, and denoising them. The speech samples were judged by 27 listeners, who all reported to be monolingual speakers of SSBE, who were raised and still living in Southern England, had not spent more than 3 years in other regions within the United Kingdom, never lived abroad, and have no knowledge of foreign languages beyond secondary school level. Listeners were recruited online, and their background requirements were verified via email.

### Experimental procedure

The experiment was conducted online, using Qualtrics XM software (www.qualtrics.com). Listeners received on-screen instructions with details about the task at hand and an incorporated audio test to check volume settings. Speech samples were presented one at a time to the listeners in semi-randomised order; that is, ensuring that listeners did not hear the sentence with the same content or the same speaker in direct succession. Three stimuli were presented twice, in order to analyse consistency of ratings. Listeners could play each sound file three times but were unable to return to previous items. For each stimulus, listeners were asked to rate the speaker’s accent as ‘native’ or ‘non-native’ (forced choice) followed by a question indicating how confident they were of their choice on a three-point scale (certain, semi-certain, or uncertain). When listeners rated a sample as non-native, they were asked to indicate which aspects of pronunciation sounded non-native to them. To give them an idea of what aspects may be involved, they were given some examples.^2^ The experiment took 65 minutes on average.

### Analysis

Following previous accent-rating experiments of L1 speech (Bergmann et al., 2016; de Leeuw et al., 2010; Mayr et al., 2020a; Moyer, 1999), the two-staged listener responses (i.e., forced choice and confidence rating) were converted into a single 6-point Likert-type scale (ranging from 1 = *certain of native-speaker status* to 6 = *certain of non-native speaker status*). A high FAR is thus indicative of a speaker who is perceived as having a strong or noticeable foreign accent in their L1 SSBE speech, whereas a low FAR is indicative of a speaker who is perceived as native or near-native, with a weak or non-existent foreign accent. First, we assessed inter-rater and intra-rater reliability by means of Fleiss’ **K**,^3^ calculated with the function *kappam.fleiss()* in the *R* package irr (Gamer et al., 2019). We then assessed whether the groups differ in their FARs by means of cumulative link mixed models (CLMMs) run in R (R Core Team, 2018) using the *ordinal* package (Christensen, 2022). CLMMs are similar to the widely used linear mixed models (LMM) but allow for the analysis of ordinal data as the dependent variable, whereas LMMs model continuous data. This CLMM had the ordinal *FARs*^4^ as the dependent variable, *Group* (i.e., *Monolingual* vs. *Bilingual*) as the fixed factor (sum-to-zero coded, i.e., with *Monolingual* = −1 and *Bilingual* = 1), and *Speaker*, *Listener*, and *Sentence* as random factors, with random slopes and intercepts for *Group* at *Listener* and *Sentence*, and random intercepts for *Group* at *Speaker*, using the formula: *FAR ~Group* *+* *(Group|Listener)* *+(Group|Sentence)* *+* *(1|Speaker)*. The anonymised data and analysis scripts for the analyses in Sections 2.5.1, 3.2.1, 3.2.2, 3.2.3, and 3.2.4 are available on the Open Science Framework at https://osf.io/zeb4f/.

Following Mayr et al. (2020a, 2020b), we used content analysis (Krippendorff, 2018) to analyse the features identified as non-native by listeners. We first screened the listener responses and removed those comments that related to aspects other than pronunciation. The remaining items were then classified as to whether they commented on segments or prosody. We subsequently classified them into more specific subcategories. For segments, these included subcategories like specific vowels or consonants, and for prosody, they included subcategories like speech/articulation rate, intonation, or stress, among others. All comments were classified by two phonetically trained raters, after which all items were checked by a third phonetically trained rater who identified possible erroneous codings or inconsistencies between the two initial raters. These were then remediated. For instance, the comment ‘Upwards inflection on Ealing with “hard l” in the middle’, was initially classified as referring to intonation, but later reclassified into two comments: one on prosody (intonation), and one on segments (/l/).

### Results

#### Accent rating

**Figure 1.**
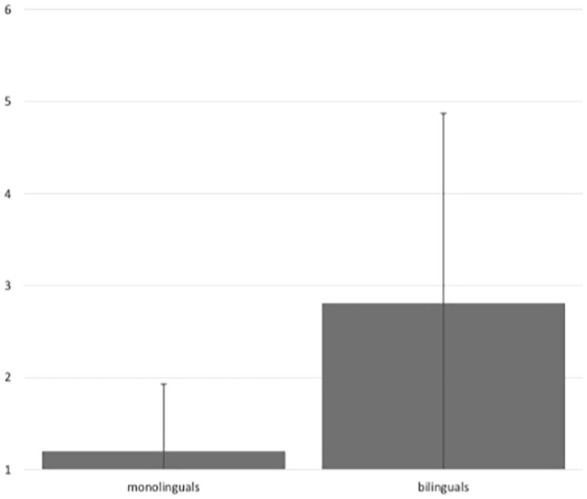
Mean FARs for monolinguals and bilinguals. Error bars represent the standard deviation.

An inter-rater reliability test revealed a Fleiss’ **K** of .25, showing a fair agreement (Landis & Koch, 1977) across the ratings made by the listeners. The intra-rater reliability test on the three repeated stimuli showed a moderate agreement in the ratings of the repeated items (Fleiss’ **K**: .56). Figure 1 shows different ratings for the monolingual (mean 1.23, *SD* .73) and bilingual (mean 2.81, *SD* 2.06) group. A CLMM confirmed that the bilingual speakers have significantly higher FARs than the monolingual speakers (χ^2^[1] = 6.9, *p* < .01).^5^

#### Perceived non-native features

Comments were provided by all listeners on all samples perceived as non-native. In total, 914 (27.7%) of the rated stimuli were perceived as non-native and thus received comments. The vast majority, that is, 886 comments involved productions by the bilingual speakers, but very occasionally (28 times) listeners also commented on stimuli by the monolingual speakers. The latter were excluded from the analysis. Following careful screening, a further 293 comments were discarded (32%), as they were too general to be classified (e.g., ‘pronunciation of *limo*’). A total of 593 comments remained. As these often mentioned two or more features, the number of feature tokens further analysed amounted to 836. A breakdown of the subcategories for the segmental features is shown in Table 1. Table 2 shows the subcategories identified for prosodic features.

**Table 1.** Perceived non-native segments.

Features identified	Tokens	% of total	% of segments	Example
Segmental features	428	51.2		
Vowels	275	32.9	64.3	
/ɪ/	60	7.2	14.0	‘ee’ sound instead of ‘i’ in ‘ni’ of *minimal*
/i**ː**/	39	4.7	9.1	lengthening of ‘e’ sound in *Ealing*
/ɔː/	17	2.0	4.0	*Multa* instead of *Malta*.
/u**ː**/	16	1.9	3.7	*you* is pronounced like ‘yuu’ instead of ‘yoo’
/eɪ/	15	1.8	3.5	*say*–closer to ‘see’ than ‘say’
/ə/	15	1.8	3.5	the ‘a’ in *phenomenal*–he pronounces like an ‘o’
/ɜ**ː**/	15	1.8	3.5	*early* is Germanic-sounding (‘uurly’)
/æ/	15	1.8	3.5	the ‘i’ instead of ‘a’ in the ‘lab’ of *syllabic*
/ɛ/	15	1.8	3.5	*best*–sounds more like ‘beyst’
/aɪ/	14	1.7	3.3	vowel in *like* was unusual
/əʊ/	9	1.1	2.1	*limo* sounded like ‘limow’
/uə/	6	0.7	1.4	*manual* pronounced differently–Manuel
/ɒ/	5	0.6	1.2	*crossword* as ‘crawsword’
/eə/	3	0.4	0.7	‘they-re’ instead of *there*
/ɑ**ː**/	3	0.4	0.7	*charge* pronounced differently to native–‘char’ was elongated
Vowels general	28	3.3	6.5	vowels all-over sound non native
Consonants	139	16.7	32.6	
/l/	38	4.5	8.9	*Ealing* with ‘hard’ ‘l’
/w/	18	2.2	4.2	*crossword* pronounced with ‘v’ instead of ‘w’
/v/	16	1.9	3.7	*leave* sounds like *leaf*
/r/	16	1.9	3.7	the r sound at the start of *remembered* sounds too soft
/z/	11	1.3	2.6	*is* is being pronounced ‘isss’ rather than ‘iz’
/ð/	11	1.3	2.6	‘th’ sound in *they* sounds more like the one in *think*
/d/	7	0.8	1.6	‘d-sounds almost sound like ‘t’s at the end
/t/	6	0.7	1.4	she’s over pronouncing the ‘t’ in *what*. I’m used to hearing a softer ‘t’.
/ŋ/	6	0.7	1.4	something about the Eali-NG sounds ‘rounded’
/ʤ/	4	0.5	0.9	‘ge’ sound in *charge* sounds like ‘ch’–‘charch’
/s/	3	0.4	0.7	*despise*–dess–longer ‘s’
/n/	2	0.2	0.5	the way they said *minimum* was different, as if the ‘n’ had an accent on it, like in Spanish
cluster	1	0.1	0.2	the ‘st’ sound in *best* sounds laboured and Germanic
Segments general	10	1.2	2.3	the pronunciation of ‘mal’ in *Malaga* and *Malta* is not right
Phoneme omission	3	0.4	0.7	*admire* pronounced differently–‘adire’–no ‘m’ sound
Phoneme insertion	2	0.2	0.5	the pronunciation of *where is* sounds like the speaker is German–the ‘is’ seems to be ‘ist’

**Table 2.** Perceived non-native prosodic features.

Features identified	Tokens	% of total	% of prosody	Example
Prosodic features	408	48.8		
Speech/articulation rate	142	17.0	34.8	They are speaking very slowly
Intonation	96	11.4	23.5	upwards inflection at the end of non-native
Stress (total)	82	9.8	20.1	
Word stress	45	5.4	11.0	*mono-SIL-abic* instead of the usual *mono-sil-Abic*
Sentence stress	25	3.0	6.1	emphasis on *puzzle* rather than *crossword*
Stress general	12	1.4	2.9	the emphasis is on different syllables than what is expected of a native speaker
Rhythm	33	3.9	8.1	rhythm across sentences seemed unusual
Syllable reduction (lack of)	41	4.9	10.0	*mi-ni-mal* split into its syllables
Loudness	2	0	0.3	quiet/hard to understand.
Prosody (general)	13	1.6	3.2	prosody on *lilies*

Inspection of the two tables shows that listener comments on segmental features only marginally outnumbered those for prosody (51.2% vs. 48.8%). For segments, vowels (32.9%) were mentioned more often than consonants (16.7%). The vowel that was commented on most was /ɪ/, where listeners always indicated it being perceived as /i**ː**/. The second most commented vowel was /i**ː**/. In this case, however, comments were more difficult to interpret and less specific (e.g., ‘vowel in *meal* pronounced in a slightly unusual way’) than for /ɪ/, with the few specific comments referring to its length (e.g., ‘vowel in *leave* was extended for longer than normal’). Comments on consonants include comments describing final devoicing in /v/, /z/ and /ʤ/ (which was mentioned specifically in all but one comment for these consonants), substitution of /w/ by /v/, and realisations of laterals and rhotics.

The most commented prosodic feature (Table 2) was speech/articulation rate, with intonation the second most commented prosodic area. Comments included reference to a slower speaking rate (e.g., ‘they are speaking very slowly’) but listeners also occasionally commented on inappropriate pausing (e.g., ‘sentence doesn’t flow very well, they momentarily pause after each word’). Comments on intonation tended to specifically refer to rising intonation in questions (in 40% of all comments on intonation), with comments such as ‘the upward inflection on Ealing makes [it] sound non-native’.

## Discussion

Study 1 aimed to gain a better understanding of the link between perceived non-nativeness in L1 speech and the features that listeners associate with their impression of non-nativeness. We conducted an accent-rating experiment where listeners judged the L1 English accent of native SSBE speakers who were long-term residents in Austria and monolingual SSBE speakers living in the United Kingdom, and then indicated which features had led them to their judgement of non-nativeness. In line with earlier work on perceived foreign accent in the L1 of bilinguals (e.g., Bergmann et al., 2016; de Leeuw et al., 2010; Hopp & Schmid, 2013; Mayr et al., 2020a), the results showed that bilinguals received significantly greater non-nativeness ratings than monolinguals.

Moreover, listeners’ impressions of non-nativeness were found to be based in equal parts on segmental and prosodic features. While this shows that perceptions of non-nativeness arise from a combination of segmental and prosodic features, it is at odds with earlier findings showing greater reliance on segments than prosody in listener judgements of perceived non-nativeness (Mayr et al., 2020a). We suspect that these differences in findings may come from differences in the stimuli used in the respective studies. Whereas Mayr et al. (2020a) used stimuli of 15-second duration, the current study used sentences of 2 to 6 seconds. It is likely that listening to shorter stimuli allows for more specific answers with listeners mentioning everything they noticed, rather than those features that stood out most. Moreover, unlike Mayr et al. (2020), whose stimuli were excerpts of semi-spontaneous speech (consisting of statements only) taken from a narrative retelling task, the stimuli used in the current study were read sentences, particularly chosen to elicit a variety of intonation patterns. Listeners were thus presented with more prosodic variation to comment upon than the stimuli in Mayr et al. (2020a). This may have increased comments on prosody. Another observation is that 41% of the listener comments in our study were on more than one accentual feature, showing that – even in short stimuli – listeners rely on multiple cues to judge a speaker’s native-speaker status. This number is higher than the 16% reported by Mayr et al. (2020a), further confirming our hypothesis that shorter stimuli allow for more specific comments and longer stimuli are more likely to result in pointing out the most salient feature. There were also fewer comments in the present study than in Mayr et al. (2020a) that could not be classified as they were too general (32% vs. 41%), which again is likely to result from differences in stimulus duration across the studies. Alternatively, the listeners in our study may have given more specific comments because they were primed to do so through the examples given of non-native speech (see Note 2).

The present investigation demonstrates that listeners are able to give detailed descriptions of accentual features of bilingual speakers which they perceived as different from monolinguals’ productions. Listeners were often very specific in their comments, referring to features that could reflect L2-induced assimilatory influences in L1 speech. Examples are comments on final devoicing, vowel tensity neutralisation of /i**ː**/ and /ɪ/, and comments on speech/articulation rate and final rising intonation. All of these may reflect an L2 influence, as Austrian German tends to devoice final consonants, shows a diminished tense-lax vowel quality difference of /i**ː**/ and /ɪ/, and is found to have a slower speech/articulation rate (Kleber et al., 2021; Mennen et al., 2023) and a higher frequency of use of final rises than is typical for SSBE (Brandstätter et al., 2016; Mennen et al., 2022; Moosmüller et al., 2015).

## Study 2: acoustic and auditory analysis of features associated with non-nativeness

The purpose of Study 2 was to test whether the features listeners associate with non-nativeness in the bilinguals’ speech actually differ from those produced by the monolingual speakers. In order to answer this question, and following Mayr et al. (2020b), we carried out an acoustic and auditory analysis of the features that were mentioned most frequently by the listeners in Study 1. For segments, the two most frequently mentioned features were the vowels /ɪ/ and /i**ː**/; for prosody, speech/articulation rate and intonation were the features most frequently commented upon.

### Method

#### Segments

The most frequently mentioned segmental features commented upon by the listeners in Study 1 related to the lax high front unrounded vowel /ɪ/ closely followed by the tense high front unrounded vowel /iː/. We therefore took acoustic measures relating to these two vowels. As the most specific comments for the tense /iː/ related to its length, we measured the duration (in ms) of all instances of /iː/ vowels occurring in stressed syllables in the stimuli of the accent-rating experiment (5 instances × 11 speakers – 2 discarded items = 53). For each speaker, the average duration for /iː/ was then expressed as a proportion of the same speaker’s average duration of the instances of /ɪ/ vowels occurring in stressed syllables in the stimuli (5 instances × 11 speakers = 55), in order to control for potential speaker-specific differences in articulation rate. These proportional durations of /iː/ were tested statistically by means of two-sample t-tests.

For /ɪ/, we expected that the listeners’ frequent comment that it sounded like /iː/ may be based on listeners perceiving a shift in the bilingual speakers’ realisation of /ɪ/ towards that of /iː/. In order to test our hypothesis of a diminished tense-lax vowel quality difference, we measured F1 and F2 in /ɪ/ and /iː/ at the temporal midpoints of the vowels in the stimuli described above. In addition, we also took F1 and F2 measures for the tense low back unrounded vowel /ɑː/ occurring in stressed syllables, taken from the larger corpus described above (2 instances × 11 speakers = 22), as this vowel did not occur in the stimuli of the accent-rating experiment but was needed as an anchor vowel (see below). Formants were calculated with Praat’s (Boersma & Weenink, 2021) procedure *To Formant (burg)*, with standard settings, that is with a search space for the first five formants between 0 Hz and 5000 Hz (for males) or 5500 Hz (for females). We then used a method of orthogonal projection (Harrington & Reubold, 2021; Stevens et al., 2019) to calculate the relative position of the target vowel /ɪ/ between the anchor vowels /iː/ and /ɑː/, where the /iː/ represents the upper left corner of each speaker’s vowel space and the /ɑː/ represents the lower right corner of a speaker’s vowel space. This allows for normalisation of the vowel spaces and quantification of the closeness of /ɪ/ and /iː/. The relative distance of the target vowel /ɪ/ to the two anchor vowels /iː/ and /ɑː/, referred to as the orthogonal-projector ratio *op* (Harrington & Reubold, 2021; Stevens et al., 2019), is obtained via the equation in (1):

(1) $$op(\vec{I})=1-2\frac{\left(\vec{I}-{\vec{c}}_{iː})\;⊙\;({\vec{c}}_{iː}-{\vec{c}}_{ɑː}\right)}{\left({\vec{c}}_{iː}-{\vec{c}}_{ɑː})\;⊙\;({\vec{c}}_{iː}-{\vec{c}}_{ɑː}\right)}$$

In this equation, $\vec{Ι}$ is the pair of values formed by the first (F1) and second (F2) formant frequencies of a given target vowel of the category [ɪ]; $\vec{c}$ and $\vec{c}$ are the centroids (means of F1 and F2 over tokens) of the two anchor vowels, respectively (i.e., of [iː] and [ɑː]); ⊙ is the scalar (inner) product of two vectors. This leads to an outcome in which a value of −1 represents the formant values corresponding to the speaker-specific means of F1 and F2, respectively, of /ɑː/, and a value of 1 represents formant values corresponding to speaker-specific means of F1 and F2 of /iː/. The midpoint between the two, that is, a value of 0, should roughly correspond to F1 and F2 of a prototypical schwa vowel. This method therefore normalises speaker-specific differences in the vowel space. Following this calculation, we tested whether the bilingual speakers differed from the monolingual speakers in their relative position of [ɪ] by means of a linear mixed effect regression model with *op* as the dependent variable, *group* (levels: *Monolingual* vs. *Bilingual*) as fixed factor, and *word* and *speaker* as random factors (with random intercepts and slopes for the *group*s).

#### Prosody

Our prosodic measures encompassed an analysis of the two most frequently mentioned features, that is, speech/articulation rate and intonation. For the analysis of speech/articulation rate, we selected all 1,252 syllables from the 132 utterances described in Section 2.2 (i.e., 12 × 8 bilinguals + 12 × 3 monolinguals = 132 utterances) and calculated the number of syllables per second. As the corpus only contained three very short pause segments in only two of the stimuli, we deleted the pauses and adjusted the time points of the following phonetic segments. Therefore, the number of syllables per second represents the so-called articulation rate (as opposed to speaking or speech rate). To test differences between monolingual and bilingual speakers in articulation rate, we applied a linear mixed model with *Articulation Rate* as a dependent variable, and *Group* (levels: *Monolingual* vs. *Bilingual*) as fixed factor; random factors were *Speaker* and *Utterance Item*, with random intercepts of *Group* for *Speaker* and random incepts and slopes of *Group* for *Utterance Item*.

For intonation, the listeners most frequently commented on final rising intonation in questions. Therefore, all questions that appeared in the accent-rating experiment were subjected to an auditory prosodic description where the presence or absence of rising intonation on the accented syllable of its last word (i.e., the nuclear syllable) was noted. This yielded a total of 6 stimuli × 11 speakers = 66 sentences coded as rising or non-rising. A Pearson’s χ^2^ test with Yates’ continuity correction was used to explore a possible effect of Speaker group (monolingual or bilingual) on the percentage of occurrence of rising versus non-rising nuclear accents.

### Results

#### High tense vowel /iː/: duration

**Figure 2.**
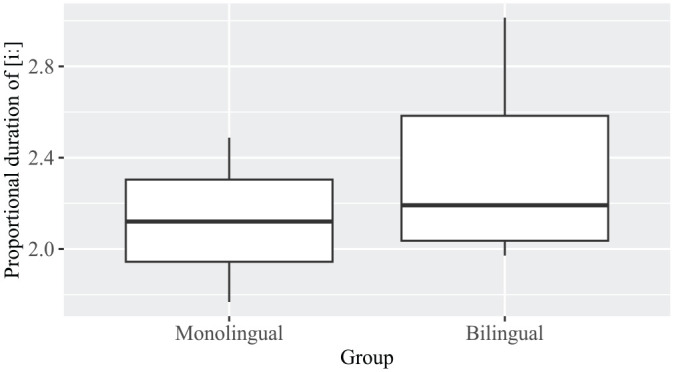
Proportional duration of [iː] vowels in monolinguals and bilinguals.

Figure 2 shows the average duration of the tense high front unrounded [iː] vowels (*N* = 56) expressed as a proportion of the average duration of the same speakers’ lax high front unrounded [ɪ] vowels (*N* = 53). No significant differences were found between the monolinguals and bilinguals, as confirmed by a two-sample *t*-test, *t*(3.9) = 0.9, n.s.

#### High lax vowel /ɪ/: closeness of /ɪ/ to /iː/

**Figure 3.**
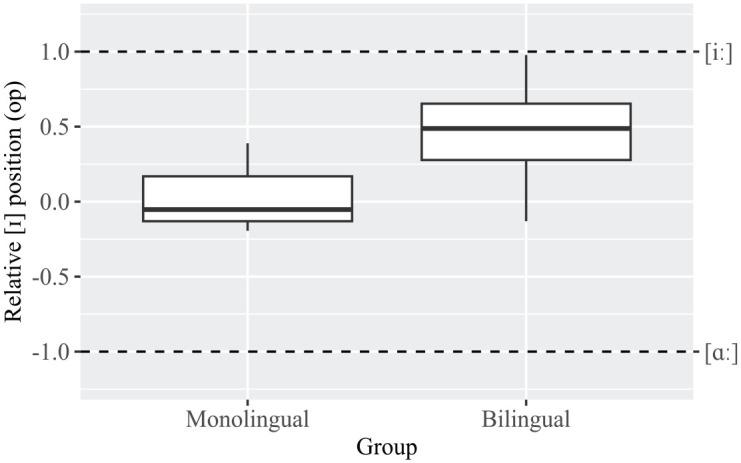
Boxplots of the *op* values for [ɪ] vowels (*N* = 62), representing their relative position in relation to [iː] (*N* = 56) and [ɑː] (*N* = 25) in monolinguals and bilinguals.

Figure 3 shows that bilinguals’ [ɪ] vowels are closer to the same speakers’ [iː] vowels compared to the monolinguals. A linear mixed model, *F*(1, 9.1) = 6.7, *p* < .05, confirms that the *op* values from equation 1 (as explained in 3.1.1), which quantify this closeness, are significantly higher in the bilinguals as opposed to the monolinguals.

#### Articulation rate

**Figure 4.**
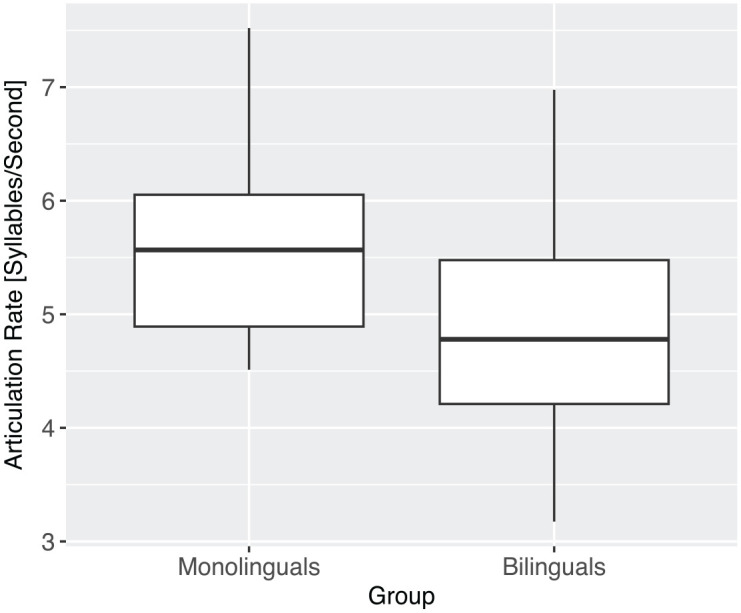
Articulation rate in monolinguals and bilinguals (*N* = 1,252 syllables).

As shown in Figure 4, bilingual speakers exhibit lower numbers (*M* = 4.9 syllables/second) for articulation rate than monolingual speakers (*M* = 5.6 syllables/second). However, a linear mixed model revealed that the observation of a slower articulation rate in the bilingual speakers just failed to reach statistical significance, *F*(1,9.5) = 4.8, n.s. (*p* = .054).

#### Final rises

**Figure 5.**
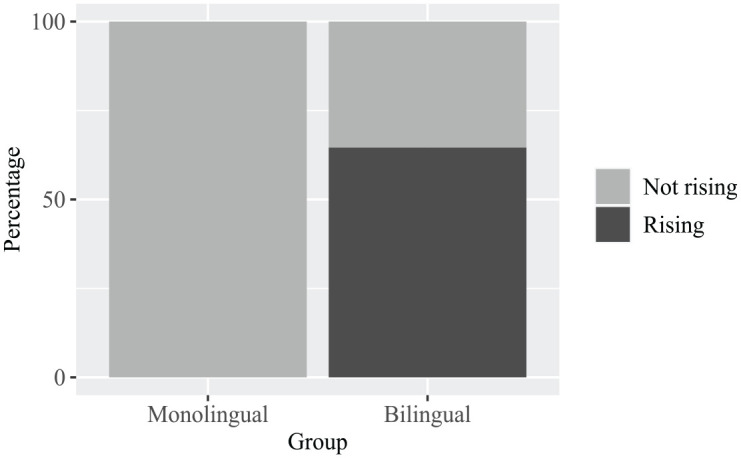
Percentage of final rises in questions produced by monolingual and bilingual speakers (*N* = 66).

Figure 5 shows differences between the monolingual and bilingual speakers in their use of rises versus falls in the final accented syllables of questions. This difference was confirmed by a Pearson’s χ^2^ test with Yates’ continuity correction, which showed an effect of *group* (BIL vs. MON), with the bilingual speakers using significantly more rises (64.6%) in final accent position in questions than the monolingual (0%) speakers (χ^2^[1] = 92.5, *p* < .001). This analysis is based on 66 nuclear accents (48 for bilinguals, 18 for monolinguals).

### Discussion

Study 2 aimed to examine whether those features that are perceived as non-native by the listeners in the accent-rating experiment (Study 1) differ from the features produced by monolingual speakers of the L1. That is, do listeners base their judgements of non-nativeness on actual differences in the stimuli they rated as non-native? Overall, the results confirmed that some of the listeners’ perceptions were indeed grounded in accentual differences in the stimuli. This was particularly the case where listener comments were very specific, as in the perceived shift of the vowel [ɪ] towards [iː], the perception in questions of ‘upward inflection at end non-native’ and the perceived slower speech rate in bilinguals, although the latter just failed to reach statistical significance. Where listeners were more variable in their comments, such as in the case of a perceived non-native length of the tense high unrounded /iː/, the listeners’ perception was not confirmed.

The most obvious source for the presence of non-native features in the English accent of bilingual speakers is the Austrian German language. In other words, it is likely that the perceived non-native features resulted from L2-induced assimilatory influences on L1 pronunciation, as predicted by the SLM (Flege, 1995; Flege & Bohn, 2021). Such assimilatory shifts of L1 sounds towards the L2 are increasingly frequently reported in the literature (e.g., Bergmann et al., 2016; de Leeuw et al., 2012, 2013; Mayr et al., 2012, 2020a; Mennen, 2004; Mennen et al., 2022; Reubold et al., 2021). For instance, Austrian German is found to have a diminished tense-lax vowel quality difference in the high front vowel space, while maintaining duration differences between /iː/ and /ɪ/ (Brandstätter et al., 2016). The fact that this was also observed in the bilinguals’ English speech samples suggests that they result from cross-language transfer. Similarly, cross-language influences are also likely to be the cause of the observed differences in the bilinguals’ frequency of use of rising intonation and their tendency to speak more slowly, as Austrian German speakers are found to use rising intonation more (Brandstätter et al., 2016; Mennen et al., 2022; Moosmüller et al., 2015) and have a slower articulation rate (Kleber et al., 2021; Mennen et al., 2023) than speakers of SSBE.

## General conclusion

The aim of the present investigation was to establish which features listeners use when determining whether individuals sound non-native in their L1, and the extent to which their impressions of non-nativeness are linked to observed accentual differences between bilingual and monolingual speakers in their production of these features. Listeners were found to rely equally on segments and prosody when judging a speaker’s native-speaker status, and were able to describe what aspects of pronunciation had created the impression of non-nativeness. Listeners referred to features that reflected L2-induced assimilatory influences in L1 speech. These features were found to correspond to actual differences in the bilinguals’ samples, originating from an influence of Austrian German on the bilinguals’ speech. In other words, listeners based their judgements of non-nativeness on assimilatory L2 influences present in the bilinguals’ L1 speech. The present investigation is hence the first to provide a direct link between the features listeners associate with perceived non-nativeness in L1 attrition contexts and the extent to which they differ from monolingual productions of these features. However, as these features show a clear influence from the L2, future studies of bilinguals with different language pairings are needed to establish whether the salience of features associated with non-nativeness is based solely on cross-language differences or whether some features are salient in all language pairs. Given that the observed differences from monolingual L1 speech patterns in our study are based on a small number of data, due to the fact that only a limited number of stimuli could be incorporated into the accent-rating experiment, future research in a larger dataset is needed. It may also be worth further examining which type and length of samples may be more successful in eliciting specific listener comments relating to differences from the monolingual L1 patterns.

In closing, the present study demonstrates that listeners are able to provide consistent, detailed, and reliable comments on what aspects of L1 speech they perceive as non-native. These accentual features were shown to reflect measurable differences from how they were realised by monolingual speakers. It can be concluded that the segmental and prosodic changes present in the L1 production of individuals undergoing attrition are clearly perceptible to native listeners and contribute to the perception of non-nativeness.

## Appendix

**Appendix 1.** Model coefficients, as given by the *R* function *summary()*, of the Cumulative Link Mixed Model *FAR ~ Group* *+* *(Group|Listener)* *+* *(Group|Sentence)* *+* *(1|Speaker)* (the factor *Group* had been sum-to-zero coded, i.e., with *Monolingual* = -1 and *Bilingual* = 1), threshold coefficients, and numbers of levels of the random factors.

Fixed effects group	*β*	*Std. error*	*z-value*	*p-value*
	1.53	0.5	3.1	< 0.01
Random effects		*Variance*	*Std. Dev.*	*Corr.*
Listener	Intercept	1.38	1.18	
	Group	0.20	0.45	.98
Sentence	Intercept	0.44	0.66	
	Group	0.10	0.31	–.698
Speaker	Intercept	2.10	1.45	
Threshold coefficients	*β*	*Std. error*	*z-value*	
1|2	1.32	0.59	2.23	
2|3	2.23	0.59	3.75	
3|4	2.53	0.59	4.27	
4|5	2.96	0.59	4.99	
5|6	4.07	0.59	6.82	
No. of levels random factors		*Listener*	*Sentence*	*Speaker*
		25	13	11

## Notes

1. The values here are derived from averages of answers to the questions ‘Please indicate to what extent you use [German|English] (a) with household members, (b) with relatives outside the household, (c) with friends outside the household, (d) with neighbours, (e) at work, (f) at school or university, (g) for public services, (h) in shops, and (i) for leisure activities’, with possible answers *never* (0), *rarely* (25), *monthly* (50), *weekly* (75), *daily* (100), and *not applicable* (NA). We then calculated the percentages of use of L1 and L2 such that their aggregate sums up to 100% for each speaker.

2. The instructions read: ‘To give you some examples, a speaker might have put emphasis on the wrong syllable and said “sub-SEE-kwent” instead of “SUB-seh-kwent”. Or, they may have mispronounced particular sounds and said < *z* > instead of < s > in “basic”, or < s > instead of <th > in “thirty”. Perhaps their pronunciation of “r” might be different than you would expect of a native speaker, or you might hear differences in the melody or rhythm of their speech. These are just some examples; you might identify different things that make a speaker sound non-native to you’.

3. Fleiss **K** assesses the inter-rater and intra-rater reliability of agreement between *N* raters, with *N* ⩾ 2. Its potential range is from 1 (‘perfect agreement’ = all answers equal) to −1 (‘perfect disagreement’ = all answers unequal).

4. That is, FARs 1 to 6, but without the assumption that the six ordered levels are necessarily equidistant from each other, as the difference between 3 = ‘uncertain of native-speaker status’ and 4 = ‘uncertain of non-native speaker status’ might be bigger than the difference between 2 and 3, which are both on the ‘native’ side of the scale.

5. See Appendix 1 for a detailed summary of the model.
